# A Novel Multiplex PCR-RFLP Method for Simultaneous Genotyping of CYP3A4*4 A>G, CYP3A4*18B G>A and CYP3A4*22 C>T

**DOI:** 10.21315/mjms2018.25.4.7

**Published:** 2018-08-30

**Authors:** Murtala Bello Abubakar, Huay Lin Tan, Siew Hua Gan

**Affiliations:** 1 Human Genome Centre, School of Medical Sciences, Universiti Sains Malaysia, 16150 Kubang Kerian, Kelantan, Malaysia; 2 Department of Physiology, Faculty of Basic Medical Sciences, College of Health Sciences, Usmanu Dan Fodiyo University PMB 2254 Sokoto, Nigeria; 3 School of Pharmacy, Monash University Malaysia, Jalan Lagoon Selatan, 47500 Bandar Sunway, Selangor, Malaysia

**Keywords:** multiplex PCR-RFLP, single nucleotide polymorphisms, CYP3A4*4, CYP3A4*18B, CYP3A4*22

## Abstract

**Background:**

Cytochrome P450 3A enzymes exhibit a variety of physiological roles and have been reported to be the most predominant enzymes involved in drugs metabolism. Single nucleotide polymorphisms (SNPs) in the genes that code for these enzymes may result in functional changes that affect enzyme activity. *CYP3A4* is an important enzyme in the metabolism of many important drugs used in the treatment of breast cancer.

**Methods:**

A total of 94 post-menopausal breast cancer patients were recruited for the study and their DNA was isolated for polymerase chain reaction (PCR). The primers were designed using Primer3 software with primer specificities checked via the Basic Local Alignment Tool (BLAST) database. The primer specificity, functionality and annealing temperature were first investigated using uniplex PCR protocols, followed by a single multiplex polymerase chain reaction-restriction fragment length polymorphism (PCR-RFLP) method. The digested amplification fragments were analysed by gel electrophoresis and subsequently validated by sequencing.

**Results:**

A multiplex PCR-RFLP method was successfully developed for simultaneous detection of CYP3A4*4, CYP3A4*18B and CYP3A4*22 in a population of post-menopausal breast cancer patients.

**Conclusion:**

The technique is simple, cost-effective, time-saving and can be routinely applied in the identification of SNPs and determination of allelic and genotypic frequencies of CYP3A4*4, CYP3A4*18B and CYP3A4*22.

## Introduction

The cytochromes P450 or commonly known as CYPs P450, are of a superfamily of heme-binding enzymes with various physiological roles (1). The cytochrome P450 3A (*CYP3A4*) is believed to be the most predominant enzyme involved in metabolism of drugs used in clinical practice (2). A significant pool of data suggests that genetic variation in the *CYP3A4* gene results in functional changes that may significantly affect its activity leading to serious consequences for patients (3, 4).

*CYP3A4* plays a key role in the metabolism of important drugs used in breast cancer treatment which include anastrozole (5), letrozole (6), exemestane (7), tamoxifen (8), cyclophosphamide, paclitaxel and docetaxel (9, 10). Genetic polymorphism in the recently described CYP3A4*22 has been shown to influence the efficacy of tamoxifen in breast cancer patients (11). A similar study also reported that breast cancer patients harbouring CYP3A4*22 had lower tendency to develop of tamoxifen-associated hot flashes (12).

Based on the updated *CYP3A4* allele nomenclature database (http://www.cypalleles.ki.se/cyp3a4.htm) the wild type of CYP3A4*1 allele category consists of subtypes CYP3A4*1A-T (2). With the exception of CYP3A4*22 and CYP3A4*18B (which are located in the intron), CYP3A4*2 to *26 alleles are found in the exons and have been reported to cause changes in protein sequences. However, only some have been reported to affect the enzyme activity in vitro (2).

CYP3A4*4 (rs55951658) located on exon 5 was previously reported in three Chinese subjects [*n* = 102] (13). The single nucleotide polymorphisms (SNP) was associated with a functionally reduced activity of the *CYP3A4* enzyme resulting in a significant lipid-lowering effects of simvastatin in hyperlipidemic patients (14) and a profound impairment of *CYP3A4* activity on endocannabinoid anadamine metabolism in vitro (15).

CYP3A4*18B (rs2242480) with a G>A SNP located in intron 10 affects cyclosporine pharmacokinetics in Chinese renal transplant recipients (16). This finding was further confirmed in healthy Chinese volunteers more recently (17). These findings suggest that CYP3A4*18B is associated with increased *CYP3A4* activity and may play a significant role in the inter-individual variability observed in cyclosporine pharmacokinetics.

CYP3A4*22 (rs35599367) with a C>T SNP located in intron 6 was recently discovered (18) and has since been established as a potentially important biomarker in drug discovery and development. The reported frequencies in Caucasians and Asians/Africans are 0.08 and 0.04, respectively (2). The presence of CYP3A4*22T-allele was further reported to be associated with midazolam clearance in renal allograft patients, indicating that there is a reduced in vivo activity of *CYP3A4* in individuals with T variant of CYP3A4*22 (19).

Inter-individual variability in drugs metabolism influences their therapeutic levels and constitutes a major concern during drug discovery and development. As highlighted above, impairment of *CYP3A4* enzyme activity due to the presence of CYP3A4*4, *18B and *22 play a significant role in this variation. This fact necessitates the need for novel therapeutic approaches geared towards improving cure rates and minimising adverse drug reactions which could be achieved by the identification of these genetic biomarkers through various pharmacogenetic studies aimed at personalised therapies. To achieve the primary goal of personalised medicine, simple, robust, fast and inexpensive methods for detection of *CYP3A4* SNPs are necessary. We report for the first time, a novel multiplex polymerase chain reactionrestriction fragment length polymorphism (PCR-RFLP) method for simultaneous detection of CYP3A4*4 A>G, CYP3A4*18B G>A and CYP3A4*22 C>T alleles.

## Materials and Methods

### Study Population and Sample Collection

This was a prospective study among post-menopausal women (aged between 44 and 83 years) with estrogen receptor positive breast cancer who attended the Oncology Clinic, Universiti Sains Malaysia, Kelantan, Malaysia. The protocol was approved by the Human Research Ethical Committee of the Universiti Sains Malaysia (USMKK/PPP/JEPeM [260.3.(21)]) which complied with the Declaration of Helsinki. The subjects were post-menopausal women [*n* = 94] with histologically confirmed hormone receptor positive stages I to III breast cancer based on the American Joint Committee on Cancer (AJCC) staging manual (sixth edition). Following the screening of the medical records, the patients were approached for study enrollment at their regular follow-up appointments. Only patients who signed written informed consents were enrolled and were then asked to complete an individual case report form. Peripheral blood (1 mL) was collected for genomic DNA extraction. The whole blood was stored in EDTA (BD Franklin Lakes, NJ USA) at −20 °C until use.

### Polymerase Chain Reaction (PCR) Method

Genomic DNA was extracted from whole blood using QIAamp® DNA Blood Mini Kit (Qiagen, Hilden, Germany) according to the manufacturer’s protocol. DNA concentration and purity were determined using Infinite® 200 NanoQuant (Tecan, Switzerland). DNA pellet was dissolved in 100 μL of TE buffer (approximately 20 ng/μL DNA concentration) and was stored at −20 °C until use.

The multiplex PCR method was developed in accordance with QIAGEN^®^ Multiplex PCR Handbook (20) using QIAGEN^®^ Multiplex PCR Plus Kit. A uniplex PCR method was first conducted to determine the specificity, functionality and annealing temperature of each primer set.

### Primer Design

The primer for amplification of CYP3A4*22 was designed using primer 3 software, version 4.0.0 (http://bioinfo.ut.ee/primer3/) (21). The primer for the amplification of CYP3A4*4 was adopted from our previous study (22) while the primer for CYP3A4*18B amplification was modified from (23). Prior to use, the primer specificity was checked using the “BLAST” database at http://blast.ncbi.nlm.nih.gov/Blast.cgi. The primer sequences are shown in Table 1.

### Hypothetical RFLP Results

The hypothetical sizes of restriction fragment length polymorphism (RFLP) for CYP3A4*4, CYP3A4*18B and CYP3A4*22 were investigated using BioEdit v7.2.5 software (http://www.mbio.ncsu.edu/bioedit/bioedit.html) and are depicted in Table 2.

### Multiplex PCR Reaction Set Up and Cycling Protocol

A total of 50 μL PCR reaction was prepared. The mixture consisted of 1× Multiplex PCR Master mix (QIAGEN^®^) containing HotStar^®^ DNA Polymerase, Multiplex PCR buffer (6 mM MgCl_2_, pH 8.7) and dNTP mix; 0.2 μM of forward and reverse primers for each SNP (*4_F, *4_R, *18B_F, *18B_R, *22_F, and *22_R), 100 ng of DNA template and double distilled water. Three samples previously confirmed by sequencing were used as positive controls in the PCR and RFLP for each of the SNP. A negative control without DNA template in the reaction mix was set up.

The cycling protocol consisted of an initial PCR activation step for 5 min at 95 °C, followed by 35 cycles of 30 s at 95 °C, 90 s at 61.9 °C and 90 s at 72 °C and a 10 min of final extension at 68 °C.

### Endonuclease Restriction Assay-RFLP Method

Prior to the RFLP, the multiplex PCR product was separated into three tubes for the genotyping of CYP3A4*4, CYP3A4*18B and CYP3A4*22 using *BsmAI* (NEB® Inc, Massachusetts, USA), *RsaI* (NEB® Inc, Massachusetts, USA) and *BseYI* (NEB® Inc, Massachusetts, USA) restriction enzymes (RE), respectively. The first tube contained 2.0 U *BsmAI*, 1× CutSmart NEBuffer^®^ (NEB® Inc, Massachusetts, USA), 0.3–0.4 μg of PCR products and 9.2 μL doubly distilled water followed by incubation at 55 °C for 60 min. (Alpha Innotech, USA). The second tube contained 4.0 U of *RsaI*, 1× CutSmart NEBuffer^®^ (NEB® Inc, Massachusetts, USA), 0.3–0.4 μg fresh PCR products and 8.8 doubly distilled water, followed by incubation at 37 °C for 60 min. The third tube contained 6.0 U *BseYI*, 1× NEBuffer 3.1^®^ (NEB® Inc, Massachusetts, USA), 0.3–0.4 μg fresh PCR template and 8.4 μL doubly distilled water; incubated at 37 °C for 60 min followed by an inactivation step at 80 °C for 20 min. The incubation for all RFLP samples was carried out using an Accublock Digital Dry Bath.

### Agarose Gel Electrophoresis

**Figure 1:**
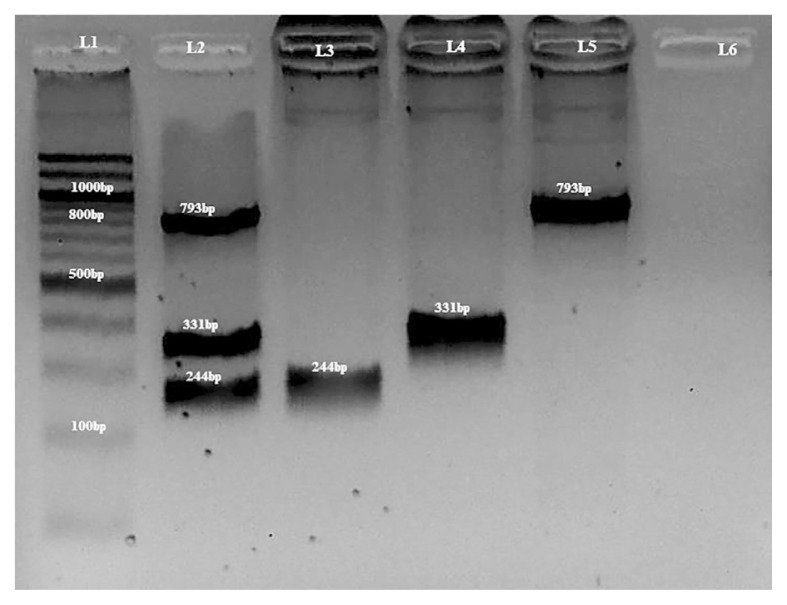
A 2% agarose gel showing PCR products from multiplex and uniplex PCR for CYP3A4*4, CYP3A4*18B and CYP3A4*22. L1: Quick-Load 100bp DNA ladder (NEB® inc, Massachusetts, USA). L2: multiplex pcr with band sizes of 244 bp, 331 bp and 793 bp. L3, L4 and L5 contain positive controls for CYP3A4*4 with 244 bp, CYP3A4*18B with 331 bp and CYP3A4*22 with 793 bp respectively. L6 is a negative control

High resolution blend agarose 3:1 HRB^TM^ gels (AMRESCO^®^, Ohio, USA) (2% and 4%) were prepared and immersed into electrophoresis gel tank containing 1× Tris-Borate-EDTA (TBE) buffer (AMRESCO^®^, Ohio, USA). For confirmation of uniplex and multiplex PCR (Figure 1), 1.0 μL of 6× DNA loading dye^®^ (Thermo Fisher Scientific Inc, Massachusetts, USA) was mixed with 1.0 μL of SYBR^®^ Green I stain (Lonza, Rockland, USA) and either 2.0 μL of a Quick-Load 100bp DNA ladder (NEB® Inc, Massachusetts, USA) or 2.0 μL of the PCR products on a Parafilm^®^ (Parafilm, Bemis, USA); the mixture was then loaded into the well and then electrophoresed on 2% agarose at 100 V for 45 min.

**Figure 2:**
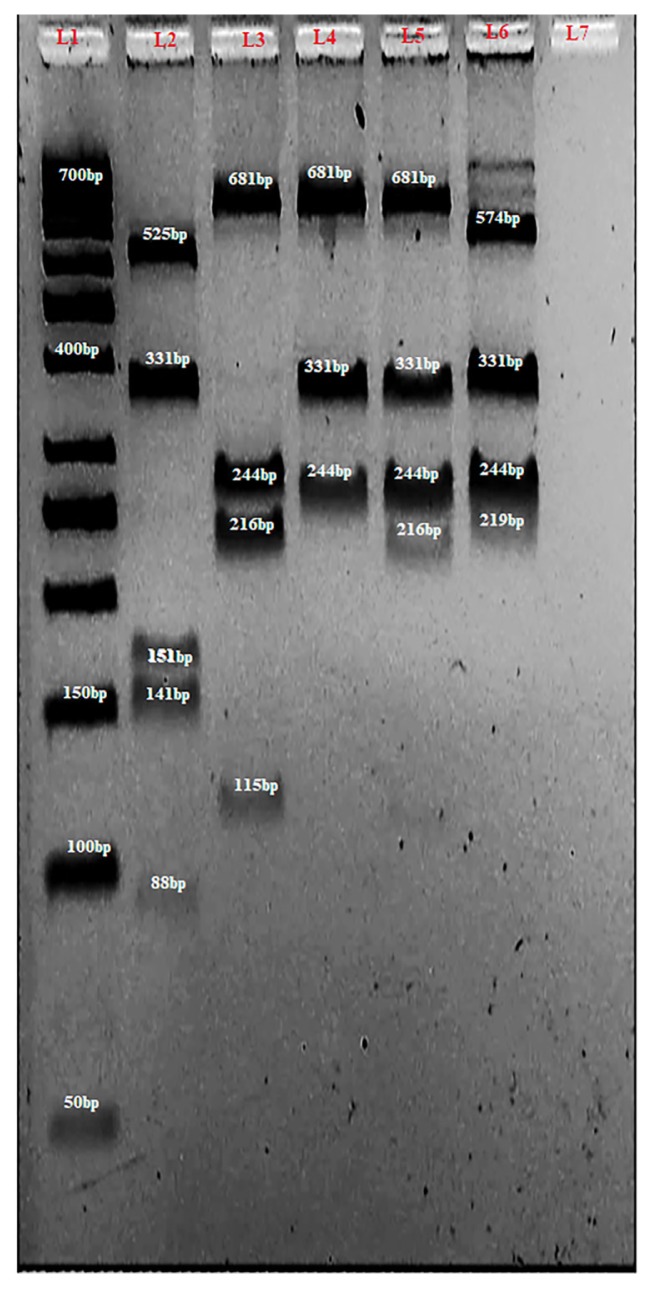
A 4% agarose gel of multiplex PCR-RFLP analysis of CYP3A4*4, CYP3A4*18B and CYP3A4*22. L1 contained GeneRuler 50bp DNA ladder (Thermo Fisher Scientific Inc, Massachusetts, USA). L2 contained wild type CYP3A4*4 allele (88 bp, and 141 bp) together with 331 bp for CYP3A4*18B and 153 bp and 525 bp from CYP3A4*22 digestions. L3, L4 and L5 contain wild type (216 bp and 115 bp), homozygous (undigested 331 bp) and heterozygous (115 bp, 216 bp and 331 bp) variants, respectively for CYP3A4*18B. They also contain 112 bp (except for L4 in which it is not shown) and 681 bp from CYP3A4*22 as well as 244 bp for CYP3A4*4. L6 contained wild type CYP3A4*22 (219 bp and 574 bp) as well as 244 bp and 331 bp for CYP3A4*4 and CYP3A4*18B, respectively. L6 contained negative control

On the other hand, for multiplex PCRRFLP (Figure 2), 1.0 μL of 6× DNA loading dye^®^ (Thermo Fisher Scientific Inc, Massachusetts, USA) was mixed with 1.0 μL of SYBR^®^ Green I stain (Lonza, Rockland, USA) and either 1.0 μL of a 50 bp DNA ladder (NEB® Inc, Massachusetts, USA) or 2.0 μL of PCR products on a Parafilm^®^ (Parafilm, Bemis, USA); the mixture was then loaded into a 4% agarose and electrophoresed at 100 V for 1.5 h.

All gels were visualised using Alpha Innotech^®^ Ultraviolet Transilluminator (Alpha Innotech^®^ USA).

### PCR Products Purification and DNA Sequencing

Prior to sequencing, the PCR products were purified using illustra^TM^ ExoProster^TM^ 1-Step Enzymatic and Sequencing Clean-Up (GE HealthCare Life Sciences, UK) according to manufacturer’s instructions.

### SNP Analysis

In order to validate our method, control samples’ nucleotide sequences were run through the snpBLAST (https://blast.ncbi.nlm.nih.gov/Blast.cgi?PAGE_TYPE=BlastSearch) and were compared against the SNP database (http://www.ensembl.org/index.html) where each SNP was identified by its reference SNP (rs) ID. For instance, rs55951658, rs2242480, and rs35599367 are the rs numbers for CYP3A4*4, CYP3A4*18B and CYP3A4*22, respectively.

## Results

In the present study, the developed multiplex PCR-RFLP method was used to successfully genotype a total of 94 patients simultaneously. The method was validated by sequencing of selected DNA samples using random sampling method (*n* = 38). For further confirmation, samples with known genotypes were run using a uniplex PCR method and the results obtained showed 100% concordance with the multiplex PCR technique (Figure 1) i.e. 244 bp, 331 bp and 793 bp for CYP3A4*4, CYP3A4*18B and CYP3A4*22, respectively.

A summary of the PCR-RFLP product sizes, endonuclease used and sizes of fragments following digestion is shown in Table 2.

### Amplification of CYP3A4*4, CYP3A4*18B and CYP3A4*22 and Their Digestions

The length of the PCR product for CYP3A4*4 was 244 bp (Figure 1). Figure 2 depicts the *BsmAI* digestion of CYP3A4*4 which yielded 15 bp (not shown), 88 bp and 141 bp for the wild type, however, no variant alleles were detected for this gene in all the 94 study subjects. In addition, the *BsmAI* also recognised the sequence *5'...G T C T C (N)_1_*↓*...3'* in CYP3A4*22 and therefore digested it to give rise to 56 bp and 59 bp (not shown), 153 bp and 525 bp. Nevertheless, the CYP3A4*18B sequence (331 bp) was unaffected by the action of *BsmAI* (Table 2 and Figure 2).

The length of the PCR product for CYP3A4*18B was 331 bp (Figure 1) and its digestion with *RsaI* is depicted in Figure 2. The *RsaI* recognises the sequence 5*'*…G T ↓ A C …3*'* and therefore the wild type CYP3A4*18B (115 bp and 216 bp) could be easily differentiated from the homozygous (undigested 331 bp) and heterozygous variants (115 bp, 216 bp and 331 bp). The *RsaI* also recognised the sequence 5*'*…G T ↓ A C …3*'* in CYP3A4*22 sequence and therefore yielded two band sizes of 112 bp and 681 bp. The CYP3A4*4 sequence (244 bp) was unaffected by the action of *RsaI* (Table 2 and Figure 2).

The length of CYP3A4*22 PCR product was 793 bp (Figure 1) and its 5*'*…C ↓ C C A G C …3*'* sequence was recognised by *BseYI* which makes it easy to differentiate the wild (219 bp and 575 bp) from the variant types. However, in this study, no variant alleles of CYP3A4*22 were detected. The CYP3A4*4 (244 bp) and CYP3A4*18B (331 bp) sequences were unaffected by the digestion with *BseYI* (Table 2 and Figure 2).

**Figure 3:**
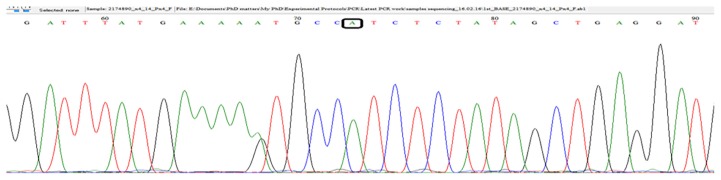
Sequencing results confirming the presence of wild type CYP3A4*4 A>G (presence of the “A” nucleotide only). The highlighted “A” is adenine indicating the absence of CYP3A4*4 SNP in this subject

**Figure 4:**
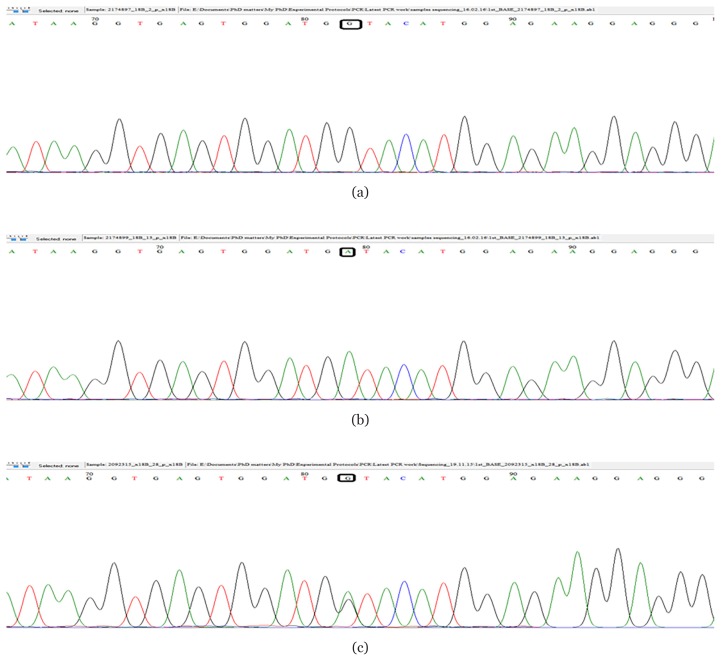
Sequencing results showing the presence of (a) wild type of CYP3A4*18B G>A; (b) homozygous variant of CYP3A4*18B G>A; (c) heterozygous variant of CYP3A4*18B G>A

**Figure 5:**
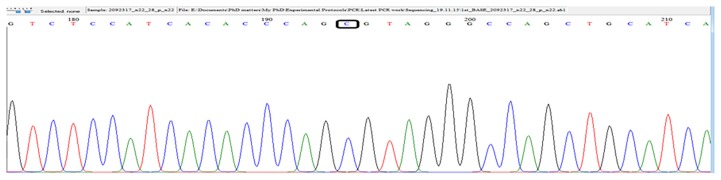
Sequencing results confirming the presence of wild type CYP3A4*22 C>T (presence of the “C” nucleotide only). The highlighted “C” is cytosine indicating the absence of CYP3A4*22 SNP in this subject

The chromatograms of *5'...G T C T C (N)_1_*↓ *...3'* for CYP3A4*4 (Figure 3)*, 5'…G T* ↓ *A C …3'* for CYP3A4*18B [Figures 4(a), 4(b) and 4(c)] and 5*'*…C ↓ C C A G C …3*'* for CYP3A4*22 (Figure 5) sequences were confirmed using a BioEdit v7.2.5 software and the sequencing results matched those of the PCR-RFLP and snpBLAST analyses.

## Discussion

Our study is the first to simultaneously determine the genotype of CYP3A4*4 A>G, CYP3A4*18B G>A and CYP3A4*22 C>T that may be useful as possible biomarkers to predict breast cancer response to treatment.

The newly developed method is stable and reproducible to be conducted in only a single-tube multiplex reaction. The method was successfully applied in genotyping of 94 subjects with a significantly minimised pre-PCR optimisation step and thermal cycling time when compared to conventional single reaction in multiple PCR tubes.

In this method, optimisation of PCR components such as MgCl_2_, dNTPs and Taq DNA polymerase was not required because the multiplex PCR master mix that was used contained pre-optimised concentrations of HotStar Taq DNA polymerase and MgCl_2_ plus dNTPs. Moreover, the multiplex PCR buffer contained a novel synthetic factor MP which enhances primer annealing and extension regardless of primer sequences. The use of a ready-made mastermix greatly reduced the time to set up the reaction while enhancing the reproducibility of the method by eliminating a variety of potential sources of pipetting errors (20).

Another critical step for a successful multiplex PCR method is primer design. There is a relationship between the primer size, its annealing temperature (T_a_) and hybridisation stability (20). Furthermore, the rule of thumb for optimum primer length is 18–30 nucleotides (24). In the present method, the length of all the primers ranged between 23–25 bases (Table 1). The melting temperature (T_m_) of a primer is the key factor in DNA-DNA hybrid stability and is important in the optimisation of a primer T_a_. In general, extremely low T_a_ can result in significant primer mispairing and the formation of multiple nonspecific bands, whereas high T_a_ may lead to the formation of insufficient primer-template hybridisation with subsequent reduction in the PCR product yield. Since the T_m_ of a primer is also related to its GC content which in turn provides information about the primers annealing stability or strength, it is recommended that each primer should have a GC content of 40%–60% (25). The present method was developed based on some of these well-established recommendations.

The use of separate tubes for the identification of CYP3A4*4 A>G, CYP3A4*18B G>A and CYP3A4*22 C>T SNPs by *BsmAI*, *RsaI,* and *BseYI,* respectively was to ensure that errors in terms of double digestion or the formation of nonspecific bands were minimised. As observed in Table 2 and Figure 2, both *BsmAI* and *RsaI* have the ability to digest the CYP3A4*22 sequence in addition to their primary targets (CYP3A4*4 and CYP3A4*18B sequences, respectively) which was unavoidable due to the long sequence of the CYP3A4*22 PCR product.

A high percentage (4%) high resolution agarose was used because of its ability to discriminate small nucleic acid fragments. Additionally the use of less-hazardous methods such as high resolution agarose is preferred over polyacrylamide which is hazardous to the central nervous system (26). Furthermore, the conventional elecptrophoresis technique involving the use of agarose gel is cost effective and is readily available for routine laboratory application.

The present method was successfully applied in the genotyping of a total of 94 breast cancer patients. Randomly selected DNA samples were sent for sequencing in order to further validate the findings. However, sequencing is believed to be more reliable than the conventional uniplex PCR-RFLP for genotyping of DNA samples (26).

A simple, rapid multiplex PCR-RFLP method will help in routine simultaneous identification of SNPs and determination of allelic and genotypic frequencies of CYP3A4*4, CYP3A4*18B and CYP3A4*22 which can be applied in various pharmacogenetics studies to predict patients’ responses to treatment and serve as a basis for personalised treatment of breast cancer as well as in many other diseases.

## Limitation of Study

The limitation of this method is that only three out of the many *CYP3A4* SNPs were simultaneously detected which was mainly due to difficulty in finding a RE that is only specific to only one sequence in each allele.

## Future Study

A multiplex method capable of simultaneous detection of more CYP3A4 SNPs is suggested in future.

## Conclusion

A simple, rapid, cost-effective and reproducible method has been successfully established for routine applications in identification of SNPs and determination of allelic and genotypic frequencies. The method does not require special equipment and requires only a small amount of standard PCR reagents.

## Figures and Tables

**Table 1:** Primer sequences for SNPs genotyping

SNPs	Primers	Sequences (5’ 3’)	Length (bp)	T_m_ (°C)	GC (%)	References
CYP3A4*4 A>G	*4_F	CACATTTTCTACAACCATGGAGACC	25	72	44.0	(22)
	*4_R	TACCTGTCCCCACCAGATTCATTCT	25	74	48.0	(22)
CYP3A4*18B G>A	*18B_F	CCACGAGCAGTGTTCTCTCCTTC	23	72	56.5	Self-designed
	*18B_R	AATAGAAAGCAGATGAACCAGAGCC	25	72	44.0	(23)
CYP3A4*22 C>T	*22_F	GCATAGAGTCTGCAGTCAGGCAAT	24	70	47.8	Self-designed
	*22_R	GATGACAGGGTTTGTGACAGGGG	23	72	56.5	Self-designed

**Table 2:** Hypothetical RFLP lengths for CYP3A4*4, CYP3A4*18B and CYP3A4*22 following digestion with *BsmAI*, *RsaI* and *BseYI*, respectively

SNPs	PCR sizes	RE and the recognition sites	REs tested	Frequencies	Fragments (bp)
CYP3A4*4	244	BsmAI			
Wild type		5'...G T C T C (N)_1_↓...3'	*BsmAI*	2	15, 88, 141
		3'...C A G A G (N)_5_↑ ...5'	*RsaI*	ND	331
			*BseYI*	ND	793
Mutant			*BsmAI*	3	15, 47, 88, 94
			*RsaI*	ND	331
			*BseYI*	ND	793

CYP3A4*18B	331	*RsaI*			
Wild type		5'…G T ↓ A C …3'	*RsaI*	1	115, 216
		3'…C A ↑ T G …5'	*BsmAI*	ND	244
			*BseYI*	ND	793
Mutant			RsaI	ND	331
			*BsmAI*	ND	244
			*BseYI*	ND	793

CYP3A4*22	793	*BseYI*			
Wild type		5'…C ↓ C C A G C …3'	*BseYI*	1	219, 574
		3' …G G G T C ↑ G …5'	*BsmAI*	3	56, 59, 153, 525
			*RsaI*	1	112, 618
Mutant			*BseYI*	ND	793
			*BsmAI*	3	56, 59, 153, 525
			*RsaI*	1	112, 681

ND: no digestion
